# Dairy consumption in adults in China: a systematic review

**DOI:** 10.1186/s40795-023-00781-2

**Published:** 2023-10-21

**Authors:** Shuhua Yang, Nupur Bhargava, Aileen O’Connor, Eileen R. Gibney, Emma L. Feeney

**Affiliations:** 1 Food for Health Ireland, University College Dublin, Dublin 4, Republic of Ireland; 2 Institute of Food and Health, University College Dublin, Dublin 4, Republic of Ireland; 3 School of Agriculture and Food Science, University College Dublin, Dublin 4, Republic of Ireland

**Keywords:** Dairy consumption, Intake, China, Systematic review

## Abstract

Research on dairy consumption in China is lacking, however, some evidence has demonstrated significant changes in recent years, with a reported increase in the overall consumption of dairy products. To fully understand these changes, a systematic review was conducted to examine reported dairy intakes and differences between dairy consumption in different population groups in China. Methods: Web of Science, Embase, and PubMed databases were searched for studies published from January 2000 to September 2022. The China National Knowledge Infrastructure (CNKI) was used to retrieve papers available in Chinese. Papers reporting dietary intakes of dairy consumption across age, sex, and geographical location sub-groups were considered for inclusion in this review. In addition, this review includes the consumption of different types of dairy foods and changes in dairy intake over time. Results: Forty-seven papers were included in the present study. Twelve papers examined dairy consumption across age groups, showing that middle-aged adults tend to consume less dairy than other age groups. Studies comparing across location-specific cohorts reported dairy intakes among urban populations were higher than rural, as well as being higher than the national average. Coastal, Northern and Eastern residents consumed more dairy products than those living in other regions of China, and people in larger cities had higher reported intakes than smaller cities. Milk was the primary dairy product reportedly consumed by Chinese population, followed by yogurt. Concerning sex, evidence showed that females generally reported a greater daily dairy intake than males. Conclusions: This review shows that, in China, several different population groups displayed significant differences in the amount and type of dairy consumed. When considering the incorporation of dairy products into healthy eating guidelines or positioning specific dairy products on the market, it is important to consider the differences and variations in consumption patterns within population groups.

## Introduction

 Dairy foods such as milk, cheese and yogurt are recognized as important sources of beneficial nutrients, including vitamins D, B5 [1] and B12 [1, 2], and minerals such as calcium [3], phosphorus, and potassium [1]. Many health benefits of dairy products are acknowledged [4], such as an impact on anthropometric measurements (i.e. weight, and waist circumference) [5, 6]. Reduced risk of hypertension (HTN) linked to dairy consumption has also been reported, whereby peptides contained within milk have been shown to reduce blood pressure through inhibition of the angiotensin pathway [7]. One study, conducted in the USA, found that each additional serving of yogurt (227 g) was associated with a 6% reduced risk of incident HTN [8]. Similarly, in a large epidemiological study of Chinese adults, a significant association between a higher frequency of dairy consumption and reduced HTN was noted [9]. Higher intake of dairy was also reported to be associated with lower blood pressure levels in a sample of Chinese young women [10]. In addition, a study in China found that regular dairy consumption (≥ 4 days/week) was associated with a lower risk of ischemic heart disease (IHD) in males [11]. Evidence has also shown that consumption of dairy may offer protection against risk of other diseases such as metabolic syndrome [12, 13], cardiovascular disease (CVD) [14–16], stroke [17], obesity [13, 18, 19], type 2 diabetes [20] and colorectal cancer [21]. However, although dairy products contain numerous beneficial nutrients, and their consumption may have a positive impact on health, there are still some concerns regarding the consumption of some dairy foods. Much of this concern is related to the saturated fatty acid (SFA) content, present in dairy products [22], known to be related to the risk of coronary heart disease (CHD) [23].

Recommendations concerning dairy consumption are given in many national nutrition and healthy eating guidelines [24–27]. In Ireland, as an example, the recommendation is 3 servings each day from the food group “milk, yoghurt and cheese” [24]. In the US, 3 daily servings of dairy products are recommended for US adults [25]. However, in Asian countries, recommendations for the consumption of dairy are lower than in western countries [28–30]. In China, a variety of dairy products, equivalent to 300ml of liquid milk per day, are recommended in the 2022 Chinese Dietary Guidelines CDGs [30].

Dietary patterns in China are known to differ quite significantly from those reported in other global regions including Europe and the US [31–35]. Traditional Chinese dietary patterns are represented by ‘Rice, vegetables, and meat’, while the ‘modern’ Chinese dietary pattern is represented by ‘fast food, milk and deep-fried food’ [34]. Similar differences are seen within the US, where two major dietary patterns has been identified from national surveys, one was ‘nonwhole grain, white potatoes, cheese, meat, discretionary oil and fat, and added sugar’, and another one was ‘whole grains, vegetable, fruits, fish, nuts and seeds’ [35]. Researchers in the US also compared Chinese dietary intakes to American diets, reporting that the Chinese diet had a lower daily intake of fiber, vitamins and some micronutrients than the American diet [33]. In China, whilst dairy products have been available and intakes of dairy have been rising in the past decades dairy consumption remains low compared to the recommended dietary guidelines for Chinese [36, 37]. This low consumption is attributed to several factors, such as lack of refrigeration, limited supply and high prices and a traditional plant-based diet [38, 39]. As a result of low intakes, in one study, dairy foods were found to contribute only 4.3% of calcium intake, with “vegetable, bean and bean products” as the main source of calcium [40]. This was relatively low compared to other countries. For instance, in Ireland, dairy contribute 38.8% of calcium to the total diet [41]. And in Poland, the contribution from dairy to total calcium intake was 54.7% in the average Polish diet [42]. However, another survey, conducted among an elderly cohort in Beijing, found that dairy products were the main contributor to calcium, contributing 34.5% among older adults aged 60 years and over [43], indicating that whilst overall consumption is low, considerable variance exists within the population.

In recent decades, the dairy industry in China has grown steadily, prompted by economic factors including the growth in household income, consumer preferences and the provision of financial support from the government [44]. However, due to existing eating habits, consumer preferences, and other historical factors such as traditional agricultural practices and dietary practices in different regions in China, variations in the consumption of dairy products exist in different sub-groups e.g. gender, location groups, which has been reported in several studies to date [45–48]. Understanding the variations in consumption may help to elucidate factors influencing intake, and support the development of strategies to increase consumption among specific population groups, in accordance with dietary recommendations [49, 50]. For instance, in the US, food based recommendations have been developed for various age and gender groups providing food choices that will help the population group to meet nutritional recommendations [50].

The purpose of this paper was to systematically review existing literature reporting dairy consumption among the Chinese population, living in mainland China. The objectives of the study were to summarise the available literature providing information on dairy intakes in the Chinese population, to examine the differences in the consumption of dairy across different population sub-groups and to further identify the factors which contribute to the differences in consumption.

## Methods

The present systematic review was carried out following the updated Preferred Reporting Items for Systematic Review and Meta-analysis (PRISMA 2020) guidelines [51]. The protocol of this review was previously registered on PROSPERO (International Prospective Register of Systematic Reviews) (registration number: CRD42021285208).

### Search strategy

Within this review, the term ‘dairy product’ is defined as milk, yogurt, milk powder, cheese, butter, cream or ice cream. The search strategy of this review followed the PICO framework, focusing on the differences in dairy consumption among different ages, geographic location sub-groups, sex groups among Chinese adults in mainland China, as well as the difference in consumption of the different types of dairy products and the overall changes in dairy consumption over time. The following search terms were used: Dairy OR Milk OR Cheese OR Yogurt OR Yoghurt OR Yoghourt OR Butter OR Cream OR Milk powder OR Food AND Intake OR Consumption OR Market OR Diet OR Dietary AND China OR Chinese OR Asian. The search was limited to studies carried out in human adults (≥ 18 years), written in English or Chinese languages. A manual search of references from included studies was also conducted. We used Google Scholar to retrieve papers where applicable. The China National Knowledge Infrastructure (CNKI) was also used to retrieve papers when the full-text papers were only available in Chinese. Two authors (S.Y. and N.B.) independently performed the literature search in Web of Science, Embase, and PubMed databases for papers published between January, 2000 and October, 2021. To ensure a focus on the most recent research regarding dairy consumption status, papers published before the year 2000 were not searched. An updated search of all the datasets was completed by one researcher (S.Y.) on 06 September 2022.

### Study screening and eligibility criteria

Published papers examining dairy intake by considering mean intake, median intake, frequency of consumption, and/or percentage of Chinese adult consumers living in mainland China were included. Study designs that were considered in this review included but not limited to dietary intake assessment study, intervention study but reporting dairy intake of control group at baseline, and consumer behaviour papers that reported findings of dairy intakes. Papers reporting the findings related to comparison of dairy consumption across age, sex, and geographical location sub-groups, different types of dairy products, and different years were included in the analysis in the present review. Papers were excluded if the original study was conducted in Chinese group living in other countries except for China. Papers were excluded if there were only children and/or teenagers involved in the study. Papers that assessed intake of human milk only were excluded. Papers reporting intakes of dairy food groups but including irrelevant food such as egg were excluded. Papers, involving intervention studies but did not report dairy intake data of participants in general good health in control group at the baseline, were excluded. For papers that reported data for those aged < 18 and ≥ 18 years, only data from those over 18 years were considered in the analysis of this review where applicable. Two authors (S.Y. and N.B.) independently screened papers for eligibility firstly based on titles, then abstracts and finally full texts based on the predefined inclusion and exclusion criteria. In the case of disagreement, a third researcher (E.R.G.) was involved, and consensus on inclusion or exclusion was reached after discussion.

### Data extraction and quality assessment

Papers included in the present review reported dairy consumption in varies ways. The following information were extracted by one author (S.Y.) firstly from the all the papers reporting dairy intakes: study characteristics (first author, publication year, sample size, study location, year of data collection, dietary assessment method); population demographic characteristics (age, sex); type of reported dairy food (total and / or individual food products if reported). For the studies using data from national survey (i.e. China Health and Nutrition Survey) without specifying study location, the survey location information was searched and taken from the survey website [52] or presented as national according to the dataset used in papers. Dietary assessment method for those papers missing relative information were taken from survey website [11] or other papers which used same survey dataset and provided more detailed information. Following, studies where they reported findings of intake differences between age groups were summarized together. Age groups in each study included in the present review were further specified and presented for the comparison within and between studies. Population size, and age details of total population and groups were displayed where applicable. Similarly, information of geographical location sub-groups, sex groups and consumption of different types of dairy products were extracted and summarized for comparison, and the changes of dairy consumption over time were also compared and presented. Basic calculation, such as counting the percentage of consumer based on the number given in papers, was conducted in this review for easier presenting and comparing of findings. Depending on the methods and analysis operated in published papers, the dairy intakes were reported in percentage of consumers, frequency of intake, mean/median intakes (g/d, kg/y, ml/d), range of intakes or descriptive sentences without statistical results in the key findings. The intake presented in this review was absolute amount of intake, not energy-adjusted. If more than one papers used data from the same study or dataset, data from the publication with the greatest detail of information were presented in this review. During the data collection, two authors (E.R.G. and E.L.F.) were involved when a paper needed to be discussed.

To assess risk of bias, the quality of the studies included in this review was examined. S.Y. performed the quality assessment. Given the various of study methods in those studies, the Critical Appraisal Skills Programme (CASP) checklist for Cohort Studies [53] was applied. The CASP checklist for Cohort Studies consists of several domains that evaluate key aspects of cohort study design, including the clarity of the research question, cohort selection, measurement of variables, consideration of confounding factors, follow-up periods, statistical analysis, and quality of results. 12 questions in the cohort study checklist was used. Two of the questions was scored up to 2 points. Total of 14 points was given if a study met all the criteria.

## Results

### Literature search results and study characteristics

**Fig. 1:**
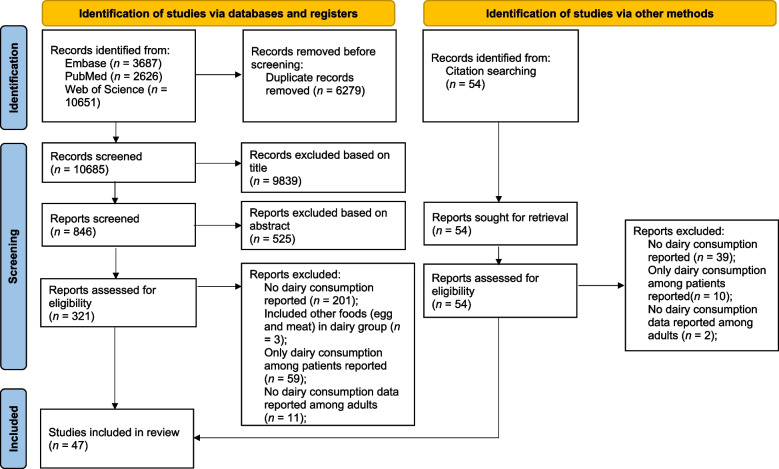
PRISMA flow diagram

A total of 10,685 papers were searched from three databases after removing duplicates. Studies identified were screened based on titles and abstracts, and finally full texts of 375 papers including the 54 papers which were identified from the reference lists were assessed according to the inclusion and exclusion criteria. Ultimately, 47 papers were included in the present study. Full details of the search are outlined in Fig. 1.

Full characteristics of the papers and CASP scores from quality assessment are shown in Table 1. Within the included papers, 24 papers reported findings on total dairy consumption. 16 papers investigated milk only. The remaining 7 papers investigated sub-groups of dairy products. Dairy intake data from 21 papers were draw from several national surveys conducted in China [46, 47, 54–72]. Within papers that reported the number of participants, sample sizes ranged from 117 to over 90,000. With respect to reported dietary intake assessment methodology, 24-hour dietary recalls [46, 47, 54–65, 67–70, 73–76], Food Frequency Questionnaires [71, 72, 77–88], Questionnaires or in-person interview [38, 45, 72, 89–92], and Internet-based dietary questionnaire for Chinese (IDQC) [93–95] were used in the data collection in reported studies to assess overall diet.

**Table 1:** Summary of studies reporting dairy intakes

Author, Year	Number of subjects	Age (years)	Sex; M%	Location of data collection	Year of data collection	Dietary assessment method	Dairy products reported	CASP score
Boyapati et al. 2003 [81]	1556	47.2 (8.7)^a^	F; N/R	Shanghai	1996 - 1998	FFQ (in-person, 5-year period)	Milk	6/14
Zhai et al. 2007 [56]	30666	18 - 45	M and F; 47.8%	Multiple cities (provinces) A	1989, 1991, 1993, 1997, 2000, 2004	24h dietary recall × 3 days	Total dairy	8/14
Fuller et al. 2007 [38]	942^b^	≤ 60	M and F; N/R	Beijing, Shanghai, Guangzhou	2001	Questionnaire (dairy only)	Milk, yogurt, milk powder, ice cream	8/14
Bai et al. 2008 [89]	638	37.95 (13.51)^a^	M and F; 50.5%	Qingdao	2005	In-person interview	Fluid milk	9/14
Zhang et al. 2008 [68]	N/R	N/R	M and F; N/R	National	1982, 1992, 2002	24h dietary recall × 1 day	Total dairy	7/14
Qiao et al. 2010 [90]	354	12 - 82	M and F; N/R	Hohhot	2008	Questionnaire (dairy only)	Milk, yogurt, milk powder, ice cream	8/14
Li et al. 2011 [67]	68962	N/R	M and F; N/R	National	2002	24h dietary recall × 1 day	Milk	7/14
Bao et al. 2012 [82]	3474	25 - 70	F; N/R	Shanghai	1996 - 1998, 2002 - 2004	FFQ (in person, 5-year period)	Milk	6/14
Fu et al. 2012 [66]	N/R	N/R	M and F; N/R	National	1990, 1995, 2000 - 2010	N/R	Total dairy	6/14
Yin et al. 2012 [71]	97187	≥ 18	M and F; 45.8%	National	2010	FFQ (in person, 1-year period)	Total dairy	9/14
Ba et al. 2013 [73]	1329	≥ 2	M and F; N/R	Beijing	N/R	24h dietary recall × 3 days	Milk	7/14
He et al. 2013 [88]	207	> 40	M and F; 41.5%	Shanghai	2008	FFQ (N/R, 1-year period)	Milk	5/14
Sun et al. 2014 [78]	20335	50 - 95	M and F; 28.8%	Guangzhou	2003 - 2006	FFQ (in-person, 1 week period)	Whole cow’s milk	8/14
Batis et al. 2014 [58]	9253	18 - 65	M and F; N/R	Multiple cities (provinces) A	1991, 1993, 1997, 2000, 2004, 2006, 2009	24h dietary recall × 3 days	Animal-based milk	7/14
Zong et al. 2014 [79]	2091	50 - 70	M and F; 41.1%	Beijing, Shanghai	2005	FFQ (N/R, N/R)	Total dairy	7/14
Tang et al. 2014 [74]	9798	≥ 40	M and F; N/R	Zhejiang	2011	24h dietary recall × 2 days	Total dairy	6/14
Fu et al. 2014 [75]	N/R	≥ 2	M and F; N/R	Hunan	1982, 1992, 2002, 2012	24h dietary recall × 3 days	Total dairy	5/14
Xu et al. 2015 [55]	2745	≥ 60	M and F; 47.4%	Multiple cities (provinces) A	2009	24h dietary recall × 3 days	Total dairy	8/14
Zhao et al. 2015 [77]	306	71.20 (7.31)^a^	M and F; 48.4%	Beijing	2013	FFQ (in person, a year period)	Milk	5/14
Zou et al. 2015 [76]	5134	≥ 18	M and F; 45.4%	Zhejiang	N/R	24h dietary recall × 1 day	Total dairy	8/14
Cheng et al. 2015 [45]	1018^b^	N/R	M and F; N/R	Beijing, Harbin	2010	In-person interview	Milk	7/14
Silanikove et al. 2015 [96]	N/R	N/R	M and F; N/R	National	2011	N/R	Milk, butter	5/14
Feng et al. 2016 [93]	292	18 - 65	M and F; 30.5%	Harbin	2014	IDQC; Diet diary × 3 days	Total dairy	7/14
Liu et al. 2016 [69]	16612	≥ 60	M and F; 49.0%	National	2010 - 2012	24h dietary recall × 3 days	Total dairy	8/14
He et al. 2016 [46]	N/R	N/R	M and F; N/R	National	1991 - 2006; 1990 - 2012; 2010 - 2012	24h dietary recall × 3 days	Milk	5/14
Song et al. 2017 [60]	11160	18 - 50	M and F; N/R	Multiple cities (provinces) B	2004, 2006, 2009, 2011	24h dietary recall × 3 days	Milk	8/14
Cheng et al. 2017 [54]	1703	18 - 75	M and F; 50.9%	Multiple cities (provinces) A	2009	24h dietary recall × 3 days	Total dairy	7/14
Shen et al. 2017 [57]	19475	≥ 18	M and F; 48.4%	Multiple cities (provinces) B	1997, 2000, 2004, 2006, 2009, 2011	24h dietary recall × 3 days	Total dairy	5/14
Tian et al. 2017 [47]	14452	42.8 (10.3)^a^	M and F; 48.1%	Multiple cities (provinces) B	2004, 2006, 2009, 2011	24h dietary recall × 3 days	Total dairy	8/14
Wang et al. 2017 [63]	4221	18 - 59	M and F; 42.9%	Multiple cities (provinces) A	1989, 1991, 1993, 1997, 2000, 2004, 2006, 2009, 2011	24h dietary recall × 3 days	Total dairy	9/14
Tian et al. 2017 [61]	5547	N/R	M and F; 49.0%	Multiple cities (provinces) B	2004, 2006, 2009, 2011	24h dietary recall × 3 days	Total dairy	5/14
Ren et al. 2018 [59]	>30000	N/R	M and F; N/R	Multiple cities (provinces) B	2011	24h dietary recall × 3 days	Total dairy	6/14
Liu et al. 2018 [70]	2552	79.42 (0.11)^a^	M and F; 50.0%	National	2010 - 2012	24h dietary record × 3days	Milk	7/14
Guo et al. 2019 [95]	6073	≥ 18	M and F; 39.1%	Northern China	2014 - 2016	IDQC	Milk, yogurt, milk powder	9/14
Huang et al. 2019 [62]	4921	≥ 60	M and F; 47.4%	Multiple cities (provinces) C	2015	24h dietary recall × 3 days	Total dairy	5/14
Song et al. 2020 [94]	3871	≥ 18	M and F; 43.9%	Dalian	2014 - 2016	IDQC	Milk, yogurt, milk powder	6/14
Zhang et al. 2020 [80]	2389	56.70 (9.99)^a^	M and F; 57.0%	Guangdong	2010 - 2018	FFQ (in person, 1 year period)	Total dairy	9/14
Cheng et al. 2020 [87]	239	44.29 (9.12)^a^	M and F; 66.1%	Sichuan	2019	FFQ (phone interview, 2 weeks period)	Total dairy	6/14
He et al. 2020 [72]	84880	60.7 (10.3)^a^	M and F; 46.4%	National	2014 - 2015	Questionnaire	Milk	7/14
Song et al. 2020 [85]	117	72.2 (8.5)^a^	M and F, 45.3%	Wuhan	2020	FFQ (online & in person, N/R)	Milk	6/14
Wang et al. 2020 [83]	18214	> 50	M and F, 29%	Guangzhou	2003 - 2006	FFQ (in person, a week period)	Milk	9/14
Wang et al. 2020 [92]	2289	27.5 (12.0)^a^	M and F, 51.4%	National	2020	Questionnaire	Total dairy	7/14
Yang et al. 2021 [91]	2702	37.3 (12.0)^a^	M and F, 29.3%	Changsha	2020	Questionnaire	Milk and Yogurt	6/14
Zhou et al. 2021 [84]	552	39 (14)^a^	M and F, 50.2%	Tibet	2020	FFQ (in person, N/R)	Total dairy	6/14
Shuai et al. 2021 [86]	1780	58 (6.0)^a^	M and F, 27.0%	Guangzhou	2008 - 2013	FFQ (in person, 1 year period)	Total dairy	7/14
Na et al. 2022 [64]	14711	42.0 (32.0, 54.0)^c^	M and F, 46.8%	Multiple cities (provinces) B	1997 - 2011	24h dietary recall × 3 days	liquid cow’s milk, yogurt, milk powder, and cheese	8/14
Wang et al. 2022 [65]	6320	≥ 60	M and F, 47.3%	Multiple cities (provinces) D	1991, 2000, 2015	24h dietary recall × 3 days	Total dairy	8/14

*F *Female, *M *Male, *FFQ *Food frequency questionnaire, *IDQC *Validated Internet-based dietary questionnaire for Chinese, *N/R *Not reported;

Multiple cities (provinces) A, Liaoning, Heilongjiang, Henan, Shandong, Hubei, Hunan, Jiangsu, Guangxi, Guizhou; Multiple cities (provinces) B, Guangxi, Guizhou, Henan, Heilongjiang, Hubei, Hunan, Jiangsu, Liaoning, and Shandong; three megacities (Beijing, Chongqing, and Shanghai) were included in 2011; Multiple cities (provinces) C, Guangxi, Guizhou, Henan, Heilongjiang, Hubei, Hunan, Jiangsu, Liaoning, Shandong, Shaanxi, Zhejiang, Yunnan, Beijing, Chongqing, and Shanghai; Multiple cities (provinces) D, Guangxi, Guizhou, Henan, Heilongjiang, Hubei, Hunan, Jiangsu, Liaoning, and Shandong; three provinces (Shaanxi, Yunnan, and Zhejiang) were added in 2015.

^a^Means and standard deviations; ^b^Number of subjects = sample means of household size × number of households; ^c^Median (interquartile range)

### Dairy consumption in different age groups

Of the 47 studies included in the final review, 12 reported dairy consumption across different age groups [45–47, 55, 62–65, 69, 73, 79, 89] (Table 2). In three studies, dairy consumption in those aged or average age over 60 were compared with other age cohorts [45, 46, 73]. Four studies focused on older cohorts aged over 50, with one reporting the differences in dairy intakes in those aged 50–70 [79], one that compared individuals aged 60–79 and 80 over [62], and three that compared ages 60–69 and 70 over [55, 65, 69]. One didn’t compare intakes between age groups but reported and compare the median age at low, high and non-consumer groups [64]. The other remaining studies included dairy consumption of working-age adults (20–59 years) [47], (18–59 years) [63], while one study used just 3 age groups to cover all ages (< 30, 30–50 and > 50) [89].

**Table 2:** Comparison of reported dairy consumption across different age groups

Author, year	Population size	Survey (location, year)	Age in total population; Age groups (years, number)	Reported intake	Key findings
Bai et al. 2008 [89]	638	Qingdao; 2005	Total population, 37.95 (13.51)^a^ Age groups^bc^, < 30 30 - 50 > 50	Milk, < 30 years: N/R 30 - 50 years: N/R > 50 years: N/R	Middle-aged (30 - 50) consumers are likely to consume less fluid milk than those aged under 30 years or over 50 years. and also less likely to be a fluid milk consumer.
Ba et al. 2013 [73]	1329^d^	Beijing; N/R	Total population, N/R Age groups^bce^, 18 - 44 45 - 59 ≥ 60	Milk, 18 - 44 years: 75.8 g/d 45 - 59 years: 96.6 g/d ≥ 60 years: 163.4 g/d	The elders had higher intakes of milk than other groups.
Zong et al. 2014 [79]	1202^f^	Beijing, Shanghai; 2005	Total population, N/R Age groups^cf^, 50 - 70, *n *= 1202	Total dairy, Amount of intake not reported.	Participants with higher dairy intake at baseline were more likely to be younger.
Xu et al. 2015 [55]	2745	Multiple cities (provinces) A; 2009	Total population, (age range: ≥ 60) Age groups^c^, 60 - 69, *n* = 1563 ≥ 70, *n* = 1182	Total dairy, (median intake, IQR) Male, 60 - 69 years: 200 g/d, 87 - 250 g/d ≥ 70 years: 162 g/d , 83 - 250 g/d Female 60 - 69 years: 167 g/d, 83 - 234 g/d ≥ 70 years: 167 g/d, 83 - 241g/d	Only 10% of older people consumed dairy. For those who consumed dairy, males aged between 60 and 69 years had higher intakes of dairy products than those aged over and 70 years.
Cheng et al. 2015 [45]	1018^d^	Beijing, Harbin; 2010	Total population, N/R Age groups^ce^, 18 - 60^b^ > 60, *n* = 43^g^	Milk, Amount of intake not reported.	Among urban residents, the elderly population (over 60 years) had higher consumption of milk compared to other residents (18 to 60 years).
He et al. 2016 [46]	N/R	N/R	Total population, N/R Age groups^bc^, 18 - 44 45 - 59 ≥ 60	Total dairy, Amount of intake not reported.	The frequency of dairy intake decreased with aging.
Liu et al. 2016 [69]	16612	National; 2010 - 2012	Total population, N/R Age groups^c^, 60 - 69, *n* = 10309 ≥ 70, *n *= 6303	Total dairy, 60 - 69 years, 28.49 g/d ≥ 70 years, 39.57 g/d	People aged 70 and over had significant higher dairy intakes than elders aged between 60 and 69. (*P* < 0.001)
Tian et al. 2017 [47]	14452	Multiple cities (provinces) B; 2004, 2006, 2009, 2011	Total population, 42.8 (10.3)^a^ Age groups^c^, 20 - 39, *n* = 5296 40 - 59, *n* = 9156	Total dairy, 20 - 39 years: 13.0 (47.1)^a^ g/d 40 - 59 years: 14.2 (55.8)^a^ g/d	People aged 40-59 had a higher consumption of dairy products compared to those aged 20 - 39, but this was not statistically significant. (*P* > 0.05)
Wang et al. 2017 [63]	4221	Multiple cities (provinces) A; 1989 - 2011	Total population, N/R Age groups^c^, 18 - 39, *n* = 1796 40 - 59, *n* = 2425	Total dairy, 18 - 39 years, 1989, 2.12 (24.28)^a^ g/d 1991, 3.03 (27.41)^a^ g/d 1993, 2.74 (24.87)^a^ g/d 1997, 3.48 (34.29)^a^ g/d 2000, 5.44 (38.75)^a^ g/d 2004, 12.49 (52.51)^a^ g/d 2006, 12.44 (72.14)^a^ g/d 2009, 10.70 (47.51)^a^ g/d 2011, 28.29 (82.02)^a^ g/d 40 - 59 years, 1989, 1.77 (19.56)^a^ g/d 1991, 4.80 (38.96)^a^ g/d 1993, 4.79 (34.86)^a^ g/d 1997, 3.16 (24.41)^a^ g/d 2000, 7.26 (38.71)^a^ g/d 2004, 15.37 (66.36)^a^ g/d 2006, 13.47 (65.34)^a^ g/d 2009, 12.07 (63.97)^a^ g/d 2011, 25.46 (84.75)^a^ g/d	The dairy consumer aged 18 - 39 had higher intakes of dairy products than people aged 40 - 59 but with no significant difference. (*P* > 0.05)
Huang et al. 2019 [62]	4921	Multiple cities (provinces)C ; 2015	Total population, N/R Age groups^c^, 60 - 79, *n* = 4490 ≥ 80, *n* = 431	Total dairy, (reported intake; percentage of consumer) 60 - 79 years, 200.0 g/d; 19.6% ≥ 80 years, 250.0 g/d; 26.2%	Elders aged 80 and over had a significant higher daily intake of dairy products than those aged 60 - 79. (*P* < 0.001); In addition, elders also had a significant higher percentage of consumers of dairy products than those aged 60 - 79. (*P* < 0.05)
Na et al. 2022 [64]	14711	Multiple cities (provinces) B; 1991, 1993, 1997, 2000, 2002, 2004, 2006, 2009, 2011	Total population, 42.0 (32.0, 54.0) Age groups^bc^,	N/R	Compare to groups of no dairy consumption and 0.1 - 100 g/d, participants in group of dairy consumption more than 100 g/d tended to be older. The median (IQR) age at baseline of the higher (> 100 g/d), lower (0.1 - 100 g/d) consumption and non-consumption groups had significant difference. (*P* < 0.001)
Wang et al. 2022 [65]	6320	Multiple cities (provinces) D; 1991, 2000, 2015	Total population, N/R Age groups^c^, 60 - 69, *n* = 3853 ≥ 70, *n* = 2467	Total dairy, 60 - 69 years, 16.3 g/d ≥ 70 years, 26.1 g/d	Older people (≥ 70) had significant higher consumption of dairy than people in group 60 - 69 years. (*P* < 0.05)

*IQR *Interquartile range, *N/R *Not reported;

Multiple cities (provinces) A, Liaoning, Heilongjiang, Henan, Shandong, Hubei, Hunan, Jiangsu, Guangxi, Guizhou; Multiple cities (provinces) B, Guangxi, Guizhou, Henan, Heilongjiang, Hubei, Hunan, Jiangsu, Liaoning, and Shandong; three municipalities of Beijing, Chongqing, and Shanghai were included in 2011; Multiple cities (provinces) C, Guangxi, Guizhou, Henan, Heilongjiang, Hubei, Hunan, Jiangsu, Liaoning, Shandong , Shaanxi, Zhejiang, Yunnan, Beijing, Chongqing, and Shanghai; Multiple cities (provinces) D, Guangxi, Guizhou, Henan, Heilongjiang, Hubei, Hunan, Jiangsu, Liaoning, and Shandong; three provinces (Shaanxi, Yunnan, and Zhejiang) were added in 2015.

^a^Mean and standard deviation; ^b^Number was not reported; ^c^Mean age was not reported; ^d^Number of total population; ^e^Age groups exclude age group < 18; ^f^Number of consumers; ^g^Number of subjects = sample means of household size × number of households.

Of the three studies that compared dairy consumption in population groups aged under and over 60 years, two of these studies showed that people aged over 60 years reported consuming higher amounts [45], while had lower frequency of milk intake [46], compared to other age groups. Ba et al. [73] found that older adults had higher intakes of milk than younger adults with daily intakes reported in older adults (≥ 60 years) of 163.4 g/d, which was significantly greater than intakes reported in those aged 18–44 years and 45–59 years, with reported milk intakes of 75.8 and 96.6 g/d, respectively.

Focusing on people aged over 50 years, dairy consumption was reported in four studies. Xu et al. [55] reported that the median dairy intakes in males aged 60–69 years who consumed dairy in 2009 was 200 g/d, while the number in males aged 70 years and over was only 162 g/d. Likewise, Zong et al. [79] found that, within the age group 50–70 years, participants with higher intakes of dairy products were more likely to be of a younger age. In addition, Liu et al. [69] and Wang et al. [65] both found that people aged 70 years and over had significantly higher dairy intakes than those aged 60–69 years (*P* < 0.001 and *P* < 0.05 separately), with average intakes in these two age groups of 39.57 and 28.49 g/d, respectively. Similarly, Huang et al. [62] compared differences in dairy consumption between the age groups 60–79 years and 80 years and over, reporting that people aged over 80 years consumed significantly more dairy. One of the largest studies, Tian et al. [47] assessed dietary intake in residents from 12 cities and provinces in 2004, 2006, 2009 and 2011, and analysed intakes across two age groups (20–39 years, 40–59 years). Within this study, those aged 40–59 years reported higher mean daily dairy intakes than those aged 20–39 years, with intakes of 14.2 ± 55.8 g/d and 13.0 ± 47.1 g/d in each age group, respectively. However, this difference was not significant (*P* > 0.05). Similarly, results from the survey of Bai et al. [89], conducted in Qingdao city in 2005, showed that the people aged over 50 years consumed more milk than other age groups. However, these differences were not statistically tested, and only reported descriptively. Additionally, Wang et al. [63] analysed the national dairy consumption data from 1989 to 2011, finding that dairy consumers aged 40–59 years had higher average dairy intakes than adults aged 18–39 years in most of the years except in 1989, 1997 and 2011. Although, this difference was not significant (*P* > 0.05).

### Dairy consumption in different geographical location groups

Of the 13 studies reporting on dairy consumption across location-specific cohorts comparing people living in different cities or provinces, two papers focused on dairy consumption in individual cities [38, 45], and eleven papers reported on dairy consumption in different regions of China classified by urban, rural; North, South, costal, inland; East, West, central; the size of city or economic status of rural area [46, 47, 56, 63–65, 67, 68, 71, 72, 79]. Table 3 summarises the characteristics and key findings of these papers.

**Table 3:** Comparison of reported dairy consumption across different geographical location groups

Author, year	Population size	Survey (location, year)	Location group (number, age)	Reported intake	Key findings
Zhai et al. 2007 [56]	30666	Multiple cities (provinces) A; 1989, 1991, 1993, 1997, 2000, 2004	Total population, (age range: 18 - 45 years) Location groups (year, number)^a^, Urban: 1989 (806), 1991 (851), 1993 (671), 1997 (717), 2000 (630), 2004 (599) Rural: 1989 (2731), 1991 (3015), 1993 (2829), 1997 (2565), 2000 (2645), 2004 (2272)	Total dairy, Total sample: 1989 (2 g/d), 1991 (4 g/d), 1993 (3 g/d), 1997 (3 g/d), 2000 (6 g/d), 2004 (12 g/d); Urban: 1989 (5 g/d), 1991 (5 g/d), 1993 (7 g/d), 1997 (9 g/d), 2000 (17 g/d), 2004 (25 g/d) Rural: 1989 (1 g/d), 1991 (2 g/d), 1993 (1 g/d), 1997 (1 g/d), 2000 (2 g/d), 2004 (6 g/d)	Milk consumption increases tended to be dominated by residents living in different locations. Urban consumers consumed more dairy than people living in rural area, and were more likely to have increased intake of milk.
Fuller et al. 2007 [38]	942^b c^	Beijing, Shanghai, Guangzhou; 2001	Total population, (age range: ≤ 60 years) Location groups^a^, North: Beijing, *n* = 300 South: Shanghai, *n* = 300, Guangzhou, *n* = 342	Milk, yogurt, milk powder, ice cream, Milk, Beijing, 56.83 kg/y Guangzhou, 27.35 kg/y Shanghai, 51.45 kg/y Yogurt, Beijing, 17.08 kg/y Guangzhou, 7.06 kg/y Shanghai, 7.64 kg/y Milk powder, Beijing, 0.84 kg/y Guangzhou, 1.14 kg/y Shanghai, 0.68 kg/y Ice cream, Beijing, 4.08 kg/y Guangzhou, 2.41 kg/y Shanghai, 0.59 kg/y	Beijing had the significant higher purchases of yogurt (*P*-value not given). Milk consumption per capita in Guangzhou was only around half of the consumption in other two cities. The consumers in Beijing had higher amount of intake of ice cream than Shanghai and Guangzhou.
Zhang et al. 2008 [68]	N/R	National; 1982, 1992, 2002	Total population, N/R Location groups^ad^, Urban Rural	Total dairy, Urban: 1982 (9.9 g/d), 1992 (36.1 g/d), 2002 (65.8 g/d); Rural: 1982 (7.3 g/d), 1992 (3.8 g/d), 2002 (11.4 g/d)	People living in urban area consumed more dairy products than people living in rural area.
Li et al. 2011 [67]	68962	National; 2002	Total population, N/R Location groups^ade^, National Urban Rural North South Coastal Inland	Milk, National: 26.60 g/d Urban: 65.80 g/d Rural: 11.40 g/d North: 33.38 g/d South: 22.24 g/d Coastal: 32.65 g/d Inland: 25.62 g/d	Compared urban residents, rural population reported lower milk intakes. People living in northern and coastal areas had higher intakes of milk than people living in south and inland respectively. (The *P*-value was not given.)
Yin et al. 2012 [71]	97187	National; 2010	Total population, (age range: ≥ 18 years) Location groups^a^, East, *n* = 14591 Central, *n* = 13838 West, *n* =16065	Total dairy, (median intake) East, 57.1 g/d Central, 35.7 g/d West, 35.7 g/d	Eastern residents had significant higher intakes of dairy products than people living in central and western. (*P *< 0.01)
Zong et al. 2014 [79]	2091	Beijing, Shanghai; 2005	Total population, (age range: 50 - 70 years) Location groups^a^, Urban, *n* = 876 Rural, *n* = 1215 North, *n* = 953 South, *n* = 1138	Total dairy, Amount of intake not reported	People from northern and urban area had higher dairy consumption.
Cheng et al. 2015 [45]	1018	Beijing, Harbin; 2010	Total population, N/R Location groups^a^, Beijing, *n* = 576 Harbin, *n* = 442	Milk, National: 13.98 kg/y Beijing : 78 kg/y Harbin : 56 kg/y	The average per capita milk consumption in Beijing is higher than in Harbin. The consumption in both cities were much higher than consumption at the national level (13.98 kg).
He et al. 2016 [46]	N/R	N/R; 2012	Total population, N/R Location groups^ad^, Big city Small and medium sized city Normal rural area Poor rural area (The classification of different areas was not given.)	Milk, Big city: 64.3 g/d Small and medium sized city: 24.2 g/d Normal rural area: 9.1 g/d Poor rural area: 4.9 g/d	There were considerable differences in milk consumption in different areas. The residents living in big cities consumed much more milk than people who lived in small or medium sized cities. Compared with citizens, people in rural areas had less intakes of milk.
Tian et al. 2017 [47]	14452	Multiple cities (provinces) B, 2004, 2006, 2009, 2011	Total population, 42.8 (10.3)^f^ years Location groups^a^, Urban, *n* = 4610 Rural, *n* = 9842	Total dairy, Urban: 30.9 (72.1)^f^ g/d Rural: 5.1 (36.7)^f^ g/d	Urban residents had significantly higher consumption of milk and its products than the rural population. (*P* < 0.05)
Wang et al. 2017 [63]	4221	Multiple cities (provinces) B; 1989, 1991, 1993, 1997, 2000, 2004, 2006, 2009, 2011	Total population, (age range: 18 - 59 years) Location groups^a^, Urban, *n* = 3231 Rural, *n* = 990	Total dairy, Urban 1989, 5.24 (38.64)^f^ g/d 1991, 9.85 (50.21)^f^ g/d 1993, 10.98 (50.90)^f^ g/d 1997, 8.74 (49.46)^f^ g/d 2000, 16.27 (62.39)^f^ g/d 2004, 32.64 (86.45)^f^ g/d 2006, 26.50 (88.06)^f^ g/d 2009, 23.98 (70.64)^f^ g/d 2011, 52.52 (115.47)^f^ g/d Rural 1989, 0.51 (9.37)^f^ g/d 1991, 0.88 (18.25)^f^ g/d 1993, 0.42 (9.51)^f^ g/d 1997, 0.59 (10.46)^f^ g/d 2000, 1.52 (16.57)^f^ g/d 2004, 4.70 (38.91)^f^ g/d 2006, 6.27 (54.02)^f^ g/d 2009, 5.36 (49.69)^f^ g/d 2011, 8.53 (43.38)^f^ g/d	There was significant difference between dairy consumption of urban and rural population in 1989-2011. (*P* < 0.0001)
He et al. 2020 [72]	84880	National; 2014 - 2015	Total population, 60.68 (10.3)^f^ years Location groups^a^, Urban, *n* = 40660 Rural, *n* = 44220	Milk, Low milk consumption Urban: *n* = 34980 (45.3%) Rural: *n* = 42242 (54.7%) High milk consumption Urban: *n* = 5680 (74.17%) Rural: *n* = 1978 (25.83%)	Between the population with low and high consumption of milk, it shows significant differences in living location (urban and rural).
Na et al. 2022 [64]	14711	Multiple cities (provinces) B; 1991, 1993, 1997, 2000, 2002, 2004, 2006, 2009, 2011	Total population, 42.0 (32.0, 54.0)^g^ Location groups^a^, East, *n* = 5616 Centra, *n* = 5450 West, *n* = 3645 Urban, *n* = 5526 Rural, *n* = 9185	Total dairy, 0.1 - 100 g/d East: *n* = 1086 (19.3%) Centra: *n* = 688 (12.6%) West: *n* = 267 (7.33%) Urban: *n* = 1386 (25.1%) Rural: *n* = 655 (7.13%) > 100 g/d East: 710 (12.6%) Centra: 69 (1.27%) West: 123 (3.37%) Urban: 742 (13.4%) Rural: 160 (1.74%)	Eastern residents had significant higher percentage of dairy consumer than central and western. (*P* < 0.001) There was significant higher percentage of dairy consumers in urban area compared to rural area. (*P *< 0.001)
Wang et al. 2022 [65]	6320	Multiple cities (provinces) D; 1991, 2000, 2015	Total population, Location groups^a^, Urban, *n* = 2482 Rural, *n* = 3838	Total dairy, Urban: 40.4 g/d Rural: 10.6 g/d	Urban residents consumed higher amount of dairy than people living in rural. (*P* < 0.05)

N/R, Not reported; Multiple cities (provinces) A, Liaoning, Heilongjiang, Henan, Shandong, Hubei, Hunan, Jiangsu, Guangxi, Guizhou; Multiple cities (provinces) B, Guangxi, Guizhou, Henan, Heilongjiang, Hubei, Hunan, Jiangsu, Liaoning, and Shandong; three municipalities of Beijing, Chongqing, and Shanghai were included in 2011; Multiple cities (provinces) D, Guangxi, Guizhou, Henan, Heilongjiang, Hubei, Hunan, Jiangsu, Liaoning, and Shandong; three provinces (Shaanxi, Yunnan, and Zhejiang) were added in 2015

^a^Mean age was not reported; ^b^Number of subjects= sample means of household size × number of households; ^c^Study includes participants aged under 18, and we were not able to separate the age group < 18 years. However, the majority of the participants were adults.; ^d^Number was not reported; ^e^The Huai River-Qinling Mountains line is generally regarded as the geographical dividing line between North China and South China. The provinces in the North of the Huai River-Qinling Mountains line were put in the North category, while these in the South were in the South category. The Coastal and Inland provinces were divided according to their location.; ^f^Mean and standard deviation; ^g^Median (interquartile range)

Nine of the 11 papers examined dairy consumption between urban and rural areas, and reported higher intakes of dairy products in urban populations compared to those living in rural areas [47, 56, 63–65, 67, 68, 72, 79]. For example, Tian et al. [47] examined milk intakes from 12 cities or provinces in 2004, 2006, 2009, 2011 in China, and reported a greater mean intake of 30.9 g/d in urban populations, compared to only 5.1 g/d in rural residents. Zhang et al. [68] reported lower mean daily dairy intakes in a rural area in 2002 of 11.4 g/d, compared to 65.8 g/d in urban residents in the same study.

Wang et al. examined differences in reported dairy intakes from urban and rural areas from 1989 to 2011 using data from CHNS. The authors reported that urban residents had a significantly higher consumption than people living in rural areas across these years (*P* < 0.0001) [63]. Most recently, He et al. reported a significant difference in high milk consumption in urban and rural areas among 31 provinces in China, with a high percentage of consumers (74.17%) are living in urban areas. The high milk consumption in this study was classified as ≥ 200 ml/day and ≥ 5 day/week [72]. In addition, one paper analyzed dietary intake data from national survey CHNS in 1991, 2000 and 2015, reporting a significant difference of mean daily intake between urban and rural residents with 40.4 g in urban areas and 10.6 g in rural areas (*P* < 0.05) [65].

Of the papers that examining dairy consumption in other geographical location groups, Li et al. [67] compared milk intakes between coastal and inland areas, reporting that people living in coastal areas had higher milk intakes than those living in inland, reporting mean intakes of 32.65 and 25.62 g/d, respectively. Research also found that those living in Northern China reported higher milk intakes than those living in Southern China in three separate studies [38, 67, 79]. For example, Li et al. [67] found that, at a national level in 2002, those in northern regions consumed more milk than people living in southern regions, with reported intakes of 33.38 g/d and 22.24 g/d, respectively. A difference in dairy consumption was also found among people living in Eastern, Central and Western areas, where it was reported that people living in Eastern cities had significant higher intakes than people living in the other two areas [64, 71]. Furthermore, only one study compared milk consumption according to the size of the city and type of rural area, demonstrating that people living in big cities consumed much more milk than those living in smaller sized cities and normal rural areas, with 64.3 g/d in big cities, 24.2 and 9.1 g/d in other areas respectively [46].

### Dairy consumption in different sex groups

Table 4 summarises the results from 16 papers that considered differences in dairy consumption across reported sex groups (male and female). All but four of these papers reported higher dairy consumption in females than males [47, 54, 55, 60, 61, 63–65, 69, 72, 78, 79, 84, 86, 92, 95]. Within those papers, eleven studies analysed data at the national level. Specifically, 8 papers analysed data from the national survey CHNS, while focusing on the different age groups and/or different collection years [47, 54, 55, 60, 61, 63–65]. One used data from the CNHS study in 2010–2012 [69]. One study analyzed the data from CNSSPP [72]. In addition, one study conducted across different regions in China [92]. The other five studies were conducted in individual cities (Beijing, Shanghai or Guangzhou) [78, 79, 86], Tibet [84] or regional locations (northern China) [95].

**Table 4:** Comparison of reported dairy consumption across different sex groups

Author, year	Population size	Survey (location, year)	Sex group (number, age)	Reported intake	Key findings
Zong et al. 2014 [79]	2091	Beijing, Shanghai; 2005	Total population, (age range: 50 - 70 years) Sex groups^a^, Male, *n* = 859 Female, *n* = 1232	Total dairy, Amount of intake not reported	Females had higher dairy consumption at study entry than males.
Sun et al. 2014 [78]	20335	Guangzhou; 2003 - 2006	Total population (age range: 50 - 95 years) Sex groups, Male, *n* = 5853, 64.7 (6.3)^b^ years, Female, *n *= 14482, 61.9 (6.7)^b^ years	Whole cow’s milk, (percentage of consuming > 250 ml/week): Male: 25% Female: 27%	Females had higher percentage of intakes of over 250 ml milk per week than males.
Xu et al. 2015 [55]	2745	Multiple cities (provinces) A; 2009	Total population (age range: ≥ 60 years) Sex groups^a^, Male, *n *= 1300 Female, *n *= 1445	Total dairy, Median (IQR) Male, 60 - 69 years: 200 g/d; 87 - 250 g/d ≥ 70 years: 162 g/d; 83 - 250 g/d, Female, 60 - 69 years: 167 g/d; 83 - 234 g/d ≥ 70 years: 167 g/d; 83 - 241 g/d	Only 10% of elders consumed dairy foods. For those who ate dairy, it showed that males consumed more dairy than females in age group 60-69. Besides, less females met the recommended intake than males for dairy, but with no statistically significant difference (*P *= 0.46).
Liu et al. 2016 [69]	16612	National; 2010 - 2012	Total population (age range: ≥ 60 years) Sex groups^a^, Male, *n *= 8148 Female, *n *= 8464	Total dairy, Male, 32.07 g/d Female, 33.03 g/d	The average intake of dairy in males was lower than females, but it’s not statistically significant. (*P* > 0.05)
Cheng et al. 2017 [54]	1703	Multiple cities (provinces) A; 2009	Total population (age range: 18 - 75 years) Sex groups, Male, *n *= 866, 44.0 (14.1)^b^ years Female, n= 837, 41.2 (12.6)^b^ years	Total dairy, Male: 9.4 (1.4)^b^ g/d Female: 17.8 (2.5)^b^ g/d	Among people in general health, females had higher consumption of dairy products than males.
Tian et al. 2017 [47]	14452	Multiple cities (provinces) B; 2004, 2006, 2009, 2011	Total population 42.8 (10.3)^b^ years Sex groups^a^, Male, *n *= 6949 Female, *n *= 7503	Total dairy, Male:11.8 (45.8)^b^ g/d Female: 15.5 (58.4)^b^ g/d	Males reported a significantly lower consumption of dairy products than female. (*P* < 0.05)
Tian et al. 2017 [61]	5547	Multiple cities (provinces) B; 2004, 2006, 2009, 2011	Total population 45.4 (11.9)^b^ years Sex groups^a^, Male, *n *= 2720 Female, *n *= 2827	Total dairy, Total sample: 13.7 (44.2)^b^ g/d Male: 12.7 (40.4)^b^ g/d Female: 14.8 (47.5)^b^ g/d	It showed that overweight females had a higher consumption of dairy than males, but not significant difference. (*P* > 0.05)
Song et al. 2017 [60]	11160	Multiple cities (provinces) B; 2004, 2006, 2009, 2011	Total population (age range: 18 - 50 years) Sex groups^ac^, Male, N/A Female, N/A	Milk, Male: 10.54 g/d Female: 13.13 g/d	Females had higher consumption of milk than males among the population of age group (18 - 50).
Wang et al. 2017 [63]	4221	Multiple cities (provinces) A; 1989, 1991, 1993, 1997, 2000, 2004, 2006, 2009, 2011	Total population (age range: 18 - 59 years) Sex groups^a^, Male, *n *= 1811 Female, *n *= 2410	Total dairy , Male 1989, 2.47 (25.42)^b^ g/d 1991, 3.88 (34.22)^b^ g/d 1993, 3.41 (28.18)^b^ g/d 1997, 3.3 (29.63)^b^ g/d 2000, 5.49 (33.47)^b^ g/d 2004, 12.20 (52.50)^b^ g/d 2006, 10.36 (45.75)^b^ g/d 2009, 10.64 (61.89)^b^ g/d 2011, 22.55 (69.93)^b^ g/d Female 1989, 1.66 (21.53)^b^ g/d 1991, 3.5 (30.07)^b^ g/d 1993, 3.69 (30.26)^b^ g/d 1997, 3.38 (30.75)^b^ g/d 2000, 7.12 (43.19)^b^ g/d 2004, 15.92 (67.56)^b^ g/d 2006, 15.56 (83.42)^b^ g/d 2009, 12.37 (54.45)^b^ g/d 2011, 29.89 (94.08)^b^ g/d	Since 1993, females had higher intakes of dairy than males, and the difference was significant. (*P *< 0.0001). However, the maximum difference was less than 7 g/d.
Guo et al. 2019 [95]	6073	Northern China; 2014 - 2016	Total population (age range: ≥ 18 years) Sex groups^a^, Male, *n *= 2375 Female, *n *= 3698	Total dairy, (percentage of males in different quartiles^d^ of mean daily intakes) Q1: 47.23%, Q2: 37.72%, Q3: 36.36%, Q4: 35.02%	It showed that there was a higher consumption of dairy in females than males. (*P* < 0.001)
He et al. 2020 [72]	84880	National; 2014 - 2015	Total population, 60.68 (10.3) ^b^ years Sex groups^a^, Male, *n *= 39365 (46.38%) Female, *n *= 45515 (53.62%)	Milk, Low consumption Male: *n *= 36276 (46.98%) Female: *n *= 40946 (53.02%) High consumption Male: *n *= 3086 (40.34%) Female: *n *= 4569 (59.66%)	In high milk consumption population, a higher percentage of females was reported.
Wang et al. 2020 [92]	2289	National; 2020	Total population 27.5 (12.0)^b^ years Sex groups, Male, *n *= 1176, 26.8 (11.9)^b^ years Females, *n *= 1113, 28.2 (12.1)^b^ years	Total dairy, Amount of intake not reported	Males reported higher frequency of dairy consumption (*P* < 0.01), and greater amount of dairy than females.
Zhou et al. 2021 [84]	552	Tibet; N/R	Total population, 39 (14)^b^ years Sex groups^a^, Male, *n *= 277 (50.2%) Female, *n *= 275 (49.8%)	Total dairy, Amount of intake not reported	Males reported higher consumption of dairy than females.
Shuai et al. 2021 [86]	1780	Guangzhou; 2008 - 2013	Total population, 58 (6.0)^b^ years Sex groups^a^, Male, *n *= 574 (32.2%) Females, *n *= 1206 (67.8%)	Total dairy, (consumers in total population) < 1 serving /month Female, 114 (52.8%) ≥ 0.5 serving /day Female, 561 (72.8%)	People with high dairy intakes were more likely to be females.
Na et al. 2022 [64]	14711	Multiple cities (provinces) B; 1991, 1993, 1997, 2000, 2002, 2004, 2006, 2009, 2011	Total population, 42.0 (32.0, 54.0)^e^ Sex groups^a^, Male, *n *= 6884 Female, *n *= 7827	Total dairy, 0.1 - 100 g/d Male, *n *= 911(13.2%) Female, *n *= 1130 (14.4%) > 100 g/d Male, *n *= 378 (5.49%) Female, *n *= 524 (6.69%)	High dairy consumption group tended to be females, and non-consumption group tended to be males. (*P* = 0.001)
Wang et al. 2022 [65]	6320	Multiple cities (provinces) D; 1991, 2000, 2015	Total population, Sex groups^a^, Male, *n *= 2987 Female, *n *= 3333	Total dairy, Male, 20.3 g/d Female, 20.2 g/d	There was no significant difference between the dairy consumption in males and females.

*IQR *Interquartile range, *N/R *Not reported; Multiple cities (provinces) A, Liaoning, Heilongjiang, Henan, Shandong, Hubei, Hunan, Jiangsu, Guangxi, Guizhou (Province); Multiple cities (provinces) B, Guangxi, Guizhou, Henan, Heilongjiang, Hubei, Hunan, Jiangsu, Liaoning, and Shandong; three municipalities of Beijing, Chongqing, and Shanghai were included in 2011; Multiple cities (provinces) D, Guangxi, Guizhou, Henan, Heilongjiang, Hubei, Hunan, Jiangsu, Liaoning, and Shandong; three provinces (Shaanxi, Yunnan, and Zhejiang) were added in 2015

^a^Mean age was not reported; ^b^Mean and standard deviation; ^c^Number was not reported; ^d^Q1 (6.42 ml/d), Q2 (39.70 ml/d), Q3 (104.01 ml/d), Q4 (227.89 ml/d) (mean); ^e^Median (interquartile range)

At the national level, papers that studied the survey data in different years from 1989 to 2011 reported higher dairy intakes among females, with significant differences found by Wang et al. [63] and Tian et al. [47], whilst the difference between sexes was either not significant or not statistically tested in other papers [54, 60, 61, 64].

Mirroring the findings from these national studies, Zong et al. [79] examined dairy consumption in males and females aged 50–70 years in Beijing and Shanghai in 2005, and found that females in this age group consumed higher amounts of dairy than males, with only 25.8% of those who consumed more than one serving of dairy foods per day being male. Sun et al. [78] collected information on milk consumption in older Chinese (aged over 50 years) in Guangzhou across two time periods ((Phase 1 (2003–2004) and Phase 2 (2005–2006)), and reported a slight difference between males and females, with 27% females and 25% males consuming over 250 ml whole cow’s milk per week, however, the results were not statistically analysed, and thus are observational. Guo et al. [95] examined the proportion of sexes across quartiles of reported dairy consumption in people living in northern China, finding that females had higher dairy intakes than males, with 47.23% males in Q1 (mean intake 6.42 ml/d), compared to 35.02% males in Q4 (mean intake 227.89 ml/d).

Four of the 16 papers examining differences in reported dairy intake across sex groups found that, for those who consumed dairy products, males had higher dairy intake compared to females with only one study reported significant difference [55, 65, 84, 92]. Xu et al., who examined reported intakes using data from CHNS 2009 [55] reported that more males met the recommended intakes for dairy than females in older adults, with median intakes in males and females aged 60–69 years 200 g/d and 167 g/d respectively. However, the differences were not statistically tested, and only provided as descriptive figures. Another study, which collected data during the COVID-19 lockdown period from March to April 2020, which examined dietary behavior across China showed that males consumed milk more frequently (*P* < 0.001) and more dairy in general compared to females [92]. Finally, another study examining intakes in the Tibetan plateau, showed greater consumption of dairy foods in males compared to females, however the amount of intake was not reported and statistically tested [84].

### Consumption of different types of dairy products

Differences in the consumption of the different types of dairy products were reported in six papers [38, 62, 90, 91, 95, 96] (Table 5). Two of the six studies reported the mean amount consumed or the range on intakes for milk, yogurt, milk powder and ice cream [38, 90]. One reported the percentage of consumers of each product among people aged 60 years and over with a focus on milk, yogurt, milk powder and other dairy products [62]. The other three focused on specific products, namely; milk, yogurt and milk powder [95], milk and yogurt [91] and only milk and butter [96].

**Table 5:** Comparison of reported consumption of different types of dairy products

Author, year	Population size and age	Survey (location, year)	Reported intake					Key findings
			Milk	Yogurt	Milk powder	Ice cream	Butter	
Fuller et al. 2007 [38]	942^a b^ Age range: ≤ 60 years	Beijing, Shanghai, Guangzhou; 2001	44.41 kg/y	10.43 kg/y	0.90 kg/y	2.36 kg/y	N/R	There was over 90 percent of households consuming milk. And the amount of milk consumption was the highest, followed by yogurt.
Qiao et al. 2010 [90]	354^b^ Age range: 12 - 82 years	Hohhot; 2008	Intake range 81.1 - 124.4 g/d	Intake range 24.5 - 33.6 g/d	Intake range 1.2 - 1.9 g/d	Intake range 5.3 - 7.6 g/d	N/R	Participants had the highest amount of milk consumption, followed by yogurt, ice cream and milk powder.
Silanikove et al. 2015 [96]	N/R	National; 2011	9.1 kg/y	N/R	N/R	N/R	0.1 kg/y	Chinese population had remarkable less consumption of butter than milk in 2011.
Huang et al. 2019 [62]	4921 Age range: ≥ 60 years	Multiple cities (provinces) C, 2015	Percentage of consumer 60 - 79, 15.6%; ≥ 80, 20.9%	Percentage of consumer 60 - 79, 5.3%; ≥ 80, 6.3%	Percentage of consumer 60 - 79, 1.0%; ≥ 80, 2.6%	N/R	N/R	Milk and yogurt were the main type of dairy products consumed by elders aged 60 and over.
Guo et al. 2019 [95]	6073 Age range: ≥ 18 years	Northern China (city or province not reported); 2014 - 2016	38.64 g/d	29.72 g/d	13.81 g/d	N/R	N/R	The amount of milk consumption ranked the highest, followed by yogurt and milk powder.
Yang et al. 2021 [91]	2702 Age range: ≥ 18 years	National; 2020	Median (IQR) 71.5 (10.7 - 150.0) ml/d	Median (IQR) 17.8 (3.6 - 71.5) ml/d	N/R	N/R	N/R	The consumption of yoghurt was much lower than milk.

*N/R *Not reported, *IQR *Interquartile range; Multiple cities (provinces) C, Guangxi, Guizhou, Henan, Heilongjiang, Hubei, Hunan, Jiangsu, Liaoning, Shandong , Shaanxi, Zhejiang, Yunnan, Beijing, Chongqing, and Shanghai

^a^Number of subjects = sample means of household size × number of households; ^b^Study includes the participants aged under 18, and we were not able to separate the age group < 18 years. However, the majority of the participants were adults

All six papers showed that participants had highest intake of milk among these types of dairy products in China. Fuller et al. [38], examining intakes in Beijing, Shanghai and Guangzhou in 2001, reported that of the annual dairy products consumed, milk consumption was highest in these three cities, with yogurt consumption ranked second, followed by ice cream and milk powder. They also reported that younger, more educated participants consumed more yogurt, whilst elderly participants tended to consume more milk powder. Similarly, the other three studies also reported much higher milk consumption than other types of dairy products (yogurt, milk powder, butter) [91, 95, 96]. Silanikove et al. [96] reported remarkably lower annual intakes of butter than milk in 2011 with 0.1 kg/y of butter and 9.1 L/y of milk. More recently, Huang et al. [62] investigated the dairy consumption in 4921 participants aged 60 years and over, and reported the percentage of consumers of each type of dairy product, finding that milk and yogurt were the main dairy products consumed in this group. Yang et al. [91] who examined the dairy consumption among adults in China during the COVID-19 lockdown, reported that the median intakes of milk and yogurt were 71.5 ml/d and 17.8 ml/d separately.

### Changes in dairy consumption over time

Seven papers report analysis of dairy consumption over time at a national level using data from CHNS [47, 56, 58, 63, 65], CNNHS [68] and NBS [66]. Of the five papers that analysed data from CHNS, one examined dairy intakes in adults aged 18–45 across 6 survey years (1989, 1991, 1993, 1997, 2000, 2004) [56], and one studied dairy intakes across four survey years (2004, 2006, 2009, 2011) among people aged 20–59 years [47]. Batis et al. [58] reported the percentage of consumers of animal-based milk during survey years 1991–2009. The other reported the dairy consumption data of adults aged 18–59 years, covering all of nine survey years (1989–2011) [63]. In addition, Wang et al. [65] examined dietary intake data in 1991, 2000 and 2015 among people aged ≥ 60 years in China. Data from these studies showed an increase in dairy intakes. For example, during the period 1989–2004, consumption of dairy products was reported to increase six-fold from 2 g/d to 12 g/d [56]. From 2004 to 2009, consumption of milk and its products then appeared to experience a decreasing trend, reaching its lowest consumption in 2009, of 25 g/d. However, from 2009 to 2011, reported intakes increased to 35 g/d, which was higher than that of the previous year [47]. Additionally, from 1991 to 2015, the average intake of dairy foods among elders had significant increase, with 8.0 g/d in 1991, 14.1 g/d in 2000 and 20.3 g/d in 2015 (*P* < 0.001) [65].Of the other two papers, Fu et al. [66] reported increasing consumption of dairy products from NBS for both urban and rural areas from 1990 to 2010, with reported dairy intakes from 0.64 kg/y to 3.55 kg/y in rural area, 4.60 kg/y to 18.10 kg/y in urban area, whereas the dairy intakes in urban residents experienced a significant decline from 22.54 to 18.10 kg/y from 2006 to 2010. The remaining paper using the data from CNNHS reported a similar increase in reported intakes of dairy products from 1982, to 1992 and 2002, reporting intakes of 8.1, 14.9, and 26.5 g/d separately [68]. It also further reported the specific changes in urban and rural areas. Compared to rural areas, urban residents reported a significantly greater increase in dairy consumption during this period, with 9.9 and 65.8 g/d reported in 1992 and 2002 in urban groups, compared to 7.3 and 11.4 g/d in rural groups. When considering differences within individual provinces, one paper reported changes in dietary intakes from 1982 to 2012 in the Hunan province, reporting that dairy intakes experienced a rapid increase from 1982 (5.9 g/d) to 2002 (95.5 g/d), but this then decreased to 16.6 g/d in 2012 [75].

In addition, researchers examined the changes of eating habits in elderly residents during COVID-19 lockdown in March 2020 in Wuhan city in China, finding that dairy consumption was reduced during this period [85]. Specifically, a 24.5% reduction was observed among males, and 45.3% among females. Considering age groups, dairy consumption reduced by 38.8% in 60–69 year old, 40.0% in 70–79 year old and 25% in those aged 80 and over.

## Discussion

Based on published literature between 2000 and 2022, which reported the consumption of total or individual dairy foods in China, some consumption patterns of dairy can be observed. Our review found noteworthy differences in dairy consumption across population groups of age, geographic location and sex, as well as differences by type of dairy. Specifically, milk and yogurt were reported to be the main dairy foods consumed in China with milk powder playing an important role in the intake of dairy in older adults. In terms of sex-related differences in dairy consumption, evidence showed that females had higher intakes than males. Clear patterns of dairy emerged across different geographical locations. The intake of dairy products among the urban population was higher than rural areas and also greater than the national average. Furthermore, coastal citizens and those in northern and eastern regions consumed more dairy products than others. Meanwhile, residents in larger cities had higher intakes than smaller cities or rural area. To the best of our knowledge, this is the first systematic review to summarise reported dairy intakes to determine factors that influence the consumption of dairy in different groups in China.

When examining dairy intake in the studies, both total dairy and also the following individual dairy foods were considered: milk, yogurt, ice cream, milk powder, butter. Much of the reporting considered total dairy and did not break down reported intakes into these individual dairy foods. From studies included in this review, milk, yogurt and milk powder were the main dairy foods reported among Chinese adults. In contrast, consumption of butter and cheese were particularly low, albeit data on these dairy products is limited. It is important to note that comparisons of reported intake of total and specific dairy products across studies are often challenging due to the manner in which dairy can be grouped and/or reported in many studies. For example, in a previous study in Poland, the main reported dairy foods were ‘Milk’, ‘Cheese and cottage cheese’, and ‘Yoghurt and milk drinks’ [97]. Similarly, a study in America grouped milk, cheese and yogurt into ‘total dairy’, excluding other dairy products [98]. In Korea, one study analysed the national data (from 2007–2009) and defined dairy products as a ‘combination of milk and yogurt’, without cheese being included, due to the extremely low consumption of cheese [99]. With such differences in the definition of dairy and grouping of dairy foods, caution must be given to comparisons across studies, since the intakes of dairy are dependent on the definition used within each study. To overcome these issues, the present review also reported on individual dairy foods when possible.

In terms of the individual dairy foods consumed, this review showed that milk was the largest contributor to dairy consumption in China, similar to other countries such as Australia [100] and Spain [101]. The present review also found that intake of yogurt was the second highest of dairy consumption, with younger and more educated consumers purchasing more yogurt than others [38]. This is different to intakes reported in other countries, where for example yogurt and fermented milk consumed among people aged 18–64 years in Spain, was less than older adults (64–75 years) [102]. In addition, data from the National Health and Nutrition Examination survey 1999–2004 in the US showed that consumption of cheese instead of yogurt ranked second among adults [103]. In contrast to western countries, we found that the consumption of cheese and butter was exceedingly low and was hardly examined in reported dairy intakes in China. One possible reason is that cheese and butter are relatively new to the market, and mostly imported, which may lead to the higher price than milk and other dairy products [44]. This may go some way to explain why consumers of these products are mostly limited to the younger and wealthier population [44]. However, more work is needed to fully understand this finding. Significant differences in the consumption of milk powder were also noticeable in the papers reported in this review. Within three identified studies reporting milk powder consumption in different survey years and locations, and among different age groups, we found that milk powder played a particular role in the diet of the Chinese population. Evidence showed that milk powder was consumed by many older adults. Before the purchase of milk and yogurt became convenient and modern refrigeration availability improved, milk powder was the most practical dairy product for consumption in China [38].

This review identified 16 papers that reported differences in dairy intakes across sex groups. Most of the available evidence showed the females had higher intakes of dairy foods than males, although not all the studies reported or conducted statistical analysis. The association between gender and dairy consumption was also observed in other recent studies examining dietary intakes in Europe [104, 105]. One study evaluated dairy intake pattern in older adults across Europe including 16 European countries, and reported that males had lower intakes of dairy than females [104]. In addition, Pellay et al. [105] analysed the socio-demographic characteristics and dietary intake among the elderly in France, finding that women were more likely to have the highest frequency of consumption of dairy foods, including milk and fresh dairy products, which also indicates that sex was a factor associated with dairy consumption. Sex has been noted as a factor which is related to dietary habits. A previous study of dietary status in China found that male participants had significantly higher consumption of vegetables, cereal, meat and legumes than females [47]. Interestingly, there was one study that reported higher dairy consumption in males than females and found that more males met the recommended intake of dairy, but these differences were not found to be significant. Since this paper didn’t give additional details of the two sex groups, we were not able to identify the reason for this result [55]. The factors that contributed to the difference of dairy consumption in females and males still need to be further investigated, but it’s clear that sex differences exist in dairy consumption in China. It is also important to note that the results in the included papers were not energy-adjusted. Therefore with the findings showing that females tend to consume higher amount of dairy than males, this need to be taken into consideration.

Associations between different regions and dairy consumption in China are considered in this present review. Based on the available papers’ comparisons across different location sub-groups including urban v rural, north south east and west, costal vs. inland, and size of city were examined. One of the main findings was that people living in urban areas had a significantly higher consumption of dairy than those living in rural areas, and this gap appears to have existed for a long time period. For example, data from a national survey in 2002 reported that the mean dairy intakes among urban residents were 65.8 g/d, whereas the amount in rural was only 11.4 g/d [67]. More recently, in 2011, the dairy intake in urban population was 52.52 ± 115.47 g/d while it was only 8.53 ± 43.38 g/d in rural area [63], suggesting no change in either of these areas. Similarly, people living in a large or even a small size city had a much higher consumption of dairy compared to those in rural areas. There are many possible reasons behind these findings such as differences in income, education level and convenience [38], which need to be explored further. People living in urban areas usually have higher incomes and are more likely to have higher education, which may have contributed to the rapid increase in consumption of dairy [44]. More supermarkets and therefore, availability of dairy products in urban areas means more choice and availability of high-quality dairy products for these population groups, which may have contributed to this difference [106]. In addition, lack of knowledge of the importance or impact of dairy products on health (or risk of disease) may also be a contributor to low dairy consumption behavior in people living in rural area [107]. The evidence also demonstrated that northern and costal populations consumed more dairy than those living in southern areas and inland cities. Compared to eastern and central regions, people living in western cities had lower dairy consumption. These differences might be due to the difference in geographic environment, food resources, social culture, and economic disparities in these regions [71]. For example, coastal and northern cities were opened to foreigners in the nineteenth century, and evidence has shown that greater exposure to western culture had a positive influence on dairy consumption [108]. Therefore, the impact of western culture on dietary patterns in those regions could be in part responsible for these differences.

Knowledge of these differences in the amount (and type) of dairy products consumed across regions, sex and age groups are of importance, as it is known that the type, and amount of dairy products consumed, can have different effects on human health [109, 110]. Dairy foods vary considerably in their nutrient compositions [109] and, evidence shows that health effects are substantially modified by the food matrix. For example, one previous study found that, dairy fat consumed in the matrix of cheese resulted in significantly lower low density lipoprotein (LDL) and total cholesterol compared with the same components eaten in the matrix of butter [110]. Many of the studies identified in the present review only considered the consumption of total dairy. The studies which did examine individual dairy foods reported considerable differences in consumption of these products within China, which merits further investigation [38, 90, 94]. We would therefore recommend that future studies capture and report details of intakes of individual dairy foods. Although dairy intakes in China have increased greatly [47], much of the data was old and more recent data was not found in published papers. With the constant change in dietary habits and more choices within food products within China, such as non-dairy plant-based milk alternatives, which are being adopted by a growing number of consumers, it is possible that a reduction of some dairy products in the Chinese population may be observed.

Whilst this review comprehensively examined the available literature, due to the complexities in reporting discussed previously, and the limited number of papers for the question being considered, the findings reported here are limited and merit further investigation. This review only presented the findings from existing comparison within the studies, therefore no analysis was conducted to compare across the studies. And there might be some published studies not identified for inclusion in this review due to the search terms used in our search. Furthermore, although limited to papers published since 2000, many of the studies use older datasets, and it is likely that dairy intakes have changed considerably and work on more recently collected data is needed. Therefore, there is a need for a detailed analysis of more recent intake data, to determine if the trends reported here are a true reflection of the current status. In addition, in this present review, we only focused on the influence of key factors - age, gender and regions which were most frequently studied and reported in published studies to investigate the difference in dairy consumption in the population group. Many other factors could be examine in future reviews.

Regardless of these limitations, this review demonstrates clear differences in consumption of different types of dairy products, and in population groups (such as males and females, age groups, urban and rural residents). When considering incorporation of dairy consumption into healthy guidelines, it is important to note these differences, and adapt recommendations and promotions accordingly. Furthermore, more detail on how dairy is specifically consumed within the diet is needed, which would support further development of nutrition recommendations through modelling scenarios for differing population groups.

## Conclusion

This review has shown deviations in dairy intake across different population groups in China, including age, sex, and geographic location as well as across the different types of dairy products. The main findings of this review demonstrate that middle-aged adults tend to consume less dairy than other age groups, females in generally had higher intakes of dairy foods than males, and that milk and yogurt and milk powder are the main types of dairy products consumed in China. Whilst this review highlighted some novel and interesting findings, it also highlights a detailed lack of understanding of the use of dairy within the diet, and differences in the dairy consumption among different population groups.

## Data Availability

The datasets used and/or analysed during the current study available from the corresponding author on reasonable request.
